# A consensus-based transparency checklist

**DOI:** 10.1038/s41562-019-0772-6

**Published:** 2019-12-02

**Authors:** Balazs Aczel, Barnabas Szaszi, Alexandra Sarafoglou, Zoltan Kekecs, Šimon Kucharský, Daniel Benjamin, Christopher D. Chambers, Agneta Fisher, Andrew Gelman, Morton A. Gernsbacher, John P. Ioannidis, Eric Johnson, Kai Jonas, Stavroula Kousta, Scott O. Lilienfeld, D. Stephen Lindsay, Candice C. Morey, Marcus Munafò, Benjamin R. Newell, Harold Pashler, David R. Shanks, Daniel J. Simons, Jelte M. Wicherts, Dolores Albarracin, Nicole D. Anderson, John Antonakis, Hal R. Arkes, Mitja D. Back, George C. Banks, Christopher Beevers, Andrew A. Bennett, Wiebke Bleidorn, Ty W. Boyer, Cristina Cacciari, Alice S. Carter, Joseph Cesario, Charles Clifton, Ronán M. Conroy, Mike Cortese, Fiammetta Cosci, Nelson Cowan, Jarret Crawford, Eveline A. Crone, John Curtin, Randall Engle, Simon Farrell, Pasco Fearon, Mark Fichman, Willem Frankenhuis, Alexandra M. Freund, M. Gareth Gaskell, Roger Giner-Sorolla, Don P. Green, Robert L. Greene, Lisa L. Harlow, Fernando Hoces de la Guardia, Derek Isaacowitz, Janet Kolodner, Debra Lieberman, Gordon D. Logan, Wendy B. Mendes, Lea Moersdorf, Brendan Nyhan, Jeffrey Pollack, Christopher Sullivan, Simine Vazire, Eric-Jan Wagenmakers

**Affiliations:** 1 ELTE, Eotvos Lorand University, Budapest, Hungary; 2 University of Amsterdam, Amsterdam, Netherlands; 3 University of Southern California, Los Angeles, CA USA; 4 Cardiff University, Cardiff, UK; 5 Columbia University, New York, NY USA; 6 University of Wisconsin-Madison, Madison, WI USA; 7 Stanford University, Stanford, CA USA; 8 Maastricht University, Maastricht, Netherlands; 9 Nature Human Behaviour, Springer Nature, London, UK; 10 Emory University, Atlanta, GA USA; 11 University of Melbourne, Melbourne, Victoria Australia; 12 University of Victoria, Saanich, British Columbia Canada; 13 University of Bristol, Bristol, UK; 14 University of New South Wales, Sydney, New South Wales Australia; 15 University of California San Diego, San Diego, CA USA; 16 University College London, London, UK; 17 University of Illinois, Chicago, IL USA; 18 Tilburg University, Tilburg, Netherlands; 19 Rotman Research Institute, Baycrest, Toronto, Ontario Canada; 20 University of Lausanne, Lausanne, Switzerland; 21 Ohio State University, Columbus, OH USA; 22 University of Münster, Münster, Germany; 23 University of North Carolina at Charlotte, Charlotte, NC USA; 24 University of Texas at Austin, Austin, TX USA; 25 Old Dominion University, Norfolk, VA USA; 26 University of California Davis, Davis, CA USA; 27 Georgia Southern University, Statesboro, GA USA; 28 University of Modena-Reggio Emilia, Modena, Italy; 29 University of Massachusetts, Boston, Boston, MA USA; 30 Michigan State University, East Lansing, MI USA; 31 University of Massachusetts, Amherst, Amherst, MA USA; 32 Royal College of Surgeons in Ireland, Dublin, Ireland; 33 University of Nebraska Omaha, Omaha, NE USA; 34 University of Florence, Florence, Italy; 35 University of Missouri, Columbia, MO USA; 36 The College of New Jersey, Ewing Township, NJ USA; 37 Leiden University, Leiden, Netherlands; 38 Georgia Institute of Technology, Atlanta, GA USA; 39 University of Western Australia, Perth, Western Australia Australia; 40 Carnegie Mellon University, New York, NY USA; 41 Radboud University, Nijmegen, Netherlands; 42 University of Zurich, Zurich, Switzerland; 43 University of York, York, UK; 44 University of Kent, Kent, UK; 45 Case Western Reserve University, Cleveland, OH USA; 46 University of Rhode Island, Providence, RI USA; 47 University of California, Berkeley, Berkeley, CA USA; 48 Northeastern University, Boston, MA USA; 49 Boston College, Boston, MA USA; 50 University of Miami, Coral Gables, FL USA; 51 Vanderbilt University, Nashville, TN USA; 52 University of California, San Francisco, San Francisco, CA USA; 53 University of Michigan, Ann Arbor, MI USA; 54 North Carolina State University, Raleigh, NC USA; 55 University of Cincinnati, Cincinnati, OH USA

**Keywords:** Scientific community, Social sciences

## Abstract

We present a consensus-based checklist to improve and document the transparency of research reports in social and behavioural research. An accompanying online application allows users to complete the form and generate a report that they can submit with their manuscript or post to a public repository.

## Good science requires transparency

Ideally, science is characterized by a ‘show me’ norm, meaning that claims should be based on observations that are reported transparently, honestly and completely^1^. When parts of the scientific process remain hidden, the trustworthiness of the associated conclusions is eroded. This erosion of trust affects the credibility not only of specific articles, but—when a lack of transparency is the norm—perhaps even entire disciplines. Transparency is required not only for evaluating and reproducing results (from the same data), but also for research synthesis and meta-analysis from the raw data and for effective replication and extension of that work. Particularly when the research is funded by public resources, transparency and openness constitute a societal obligation.

In recent years many social and behavioural scientists have expressed a lack of confidence in some past findings^2^, partly due to unsuccessful replications. Among the causes for this low replication rate are underspecified methods, analyses and reporting practices. These research practices can be difficult to detect and can easily produce unjustifiably optimistic research reports. Such lack of transparency need not be intentional or deliberately deceptive. Human reasoning is vulnerable to a host of pernicious and often subtle biases, such as hindsight bias, confirmation bias and motivated reasoning, all of which can drive researchers to unwittingly present a distorted picture of their results.

## The practical side of transparency

How can scientists increase the transparency of their work? To begin with, they could adopt open research practices such as study preregistration and data sharing^3–5^. Many journals, institutions and funders now encourage or require researchers to adopt these practices. Some scientific subfields have seen broad initiatives to promote transparency standards for reporting and summarizing research findings, such as START, SPIRIT, PRISMA, STROBE and CONSORT (see https://www.equator-network.org). A few journals ask authors to answer checklist questions about statistical and methodological practices (e.g., the Nature Life Sciences Reporting Summary)^6^ and transparency (for example, *Psychological Science*). Journals can signal that they value open practices by offering ‘badges’ that acknowledge open data, code and materials^7^. The Transparency and Openness Promotion (TOP) guidelines^8^, endorsed by many journals, promote the availability of all research items, including data, materials and code. Authors can declare their adherence to these TOP standards by adding a transparency statement in their articles (TOP Statement)^9^. Collectively, these somewhat piecemeal innovations illustrate a science-wide shift toward greater transparency in research reports.

## Transparency Checklist

We provide a consensus-based, comprehensive transparency checklist that behavioural and social science researchers can use to improve and document the transparency of their research, especially for confirmatory work. The checklist reinforces the norm of transparency by identifying concrete actions that researchers can take to enhance transparency at all the major stages of the research process. Responses to the checklist items can be submitted along with a manuscript, providing reviewers, editors and, eventually, readers with critical information about the research process necessary to evaluate the robustness of a finding. Journals could adopt this checklist as a standard part of the submission process, thereby improving documentation of the transparency of the research that they publish.

We developed the checklist contents using a preregistered ‘reactive-Delphi’ expert consensus process^10^, with the goal of ensuring that the contents cover most of the elements relevant to transparency and accountability in behavioural research. The initial set of items was evaluated by 45 behavioural and social science journal editors-in-chief and associate editors, as well as 18 open-science advocates. The Transparency Checklist was iteratively modified by deleting, adding and rewording the items until a sufficiently high level of acceptability and consensus were reached and no strong counter arguments for single items were made (for the selection of the participants and the details of the consensus procedure see Supplementary Information). As a result, the checklist represents a consensus among these experts.

The final version of the Transparency Checklist 1.0 contains 36 items that cover four components of a study: preregistration; methods; results and discussion; and data, code and materials availability. For each item, authors select the appropriate answer from prespecified options. It is important to emphasize that none of the responses on the checklist is a priori good or bad and that the transparency report provides researchers the opportunity to explain their choices at the end of each section.

**Fig. 1:**
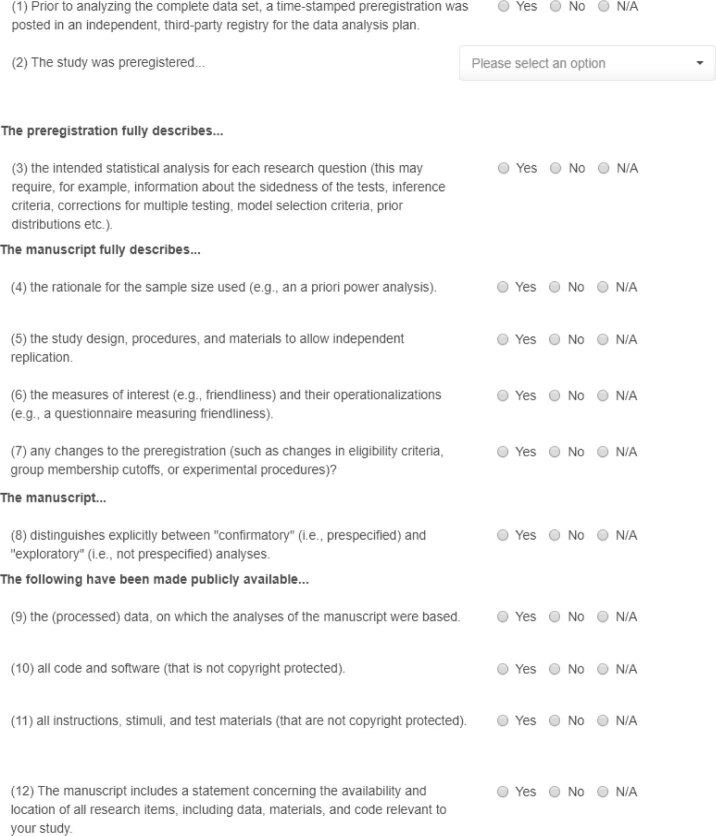
The Shortened Transparency Checklist 1.0. After each section, the researchers can add free text if they find that further explanation of their response is needed. The full version of the checklist can be found at http://www.shinyapps.org/apps/TransparencyChecklist/.

In addition to the full checklist, we provide a shortened 12-item version (Fig. 1). By reducing the demands on researchers’ time to a minimum, the shortened list may facilitate broader adoption, especially among journals that intend to promote transparency but are reluctant to ask authors to complete a 36-item list. We created online applications for the two checklists that allow users to complete the form and generate a report that they can submit with their manuscript and/or post to a public repository (Box 1). The checklist is subject to continual improvement, and users can always access the most current version on the checklist website; access to previous versions will be provided on a subpage.

This checklist presents a consensus-based solution to a difficult task: identifying the most important steps needed for achieving transparent research in the social and behavioural sciences. Although this checklist was developed for social and behavioural researchers who conduct and report confirmatory research on primary data, other research approaches and disciplines might find value in it and adapt it to their field’s needs. We believe that consensus-based solutions and user-friendly tools are necessary to achieve meaningful change in scientific practice. While there may certainly remain important topics the current version fails to cover, nonetheless we trust that this version provides a useful to facilitate starting point for transparency reporting. The checklist is subject to continual improvement, and we encourage researchers, funding agencies and journals to provide feedback and recommendations. We also encourage meta-researchers to assess the use of the checklist and its impact in the transparency of research.

Box 1 Online applications and the benefits of the transparency checklist **Online applications for the checklist**

- http://www.shinyapps.org/apps/TransparencyChecklist/ for the complete, 36-item version
- http://www.shinyapps.org/apps/ShortTransparencyChecklist/ for the shortened, 12-item version

**Benefits of the checklist**

- The checklist can help authors improve the transparency of their work before submission.
- Disclosed checklist responses can help editors, reviewers and readers gain insight into the transparency of the submitted studies.
- Guidelines built on the checklist can be used for educational purposes and to raise the standards of social and behavioural sciences, as well as other scientific disciplines, regarding transparency and credibility.
- Funding agencies can use a version of this checklist to improve the research culture and accelerate scientific progress.

## Supplementary information

Supplementary Materials Supplementary Methods, Supplementary Figure 1, Supplementary Table 1.

## Data Availability

All anonymized raw and processed data as well as the survey materials are publicly shared on the Open Science Framework page of the project: https://osf.io/v5p2r/. Our methodology and data-analysis plan were preregistered before the project. The preregistration document can be accessed at: https://osf.io/v5p2r/registrations.
