# Author Correction: A consensus-based transparency checklist

**DOI:** 10.1038/s41562-019-0812-2

**Published:** 2019-12-23

**Authors:** Balazs Aczel, Barnabas Szaszi, Alexandra Sarafoglou, Zoltan Kekecs, Šimon Kucharský, Daniel Benjamin, Christopher D. Chambers, Agneta Fisher, Andrew Gelman, Morton A. Gernsbacher, John P. Ioannidis, Eric Johnson, Kai Jonas, Stavroula Kousta, Scott O. Lilienfeld, D. Stephen Lindsay, Candice C. Morey, Marcus Munafò, Benjamin R. Newell, Harold Pashler, David R. Shanks, Daniel J. Simons, Jelte M. Wicherts, Dolores Albarracin, Nicole D. Anderson, John Antonakis, Hal R. Arkes, Mitja D. Back, George C. Banks, Christopher Beevers, Andrew A. Bennett, Wiebke Bleidorn, Ty W. Boyer, Cristina Cacciari, Alice S. Carter, Joseph Cesario, Charles Clifton, Ronán M. Conroy, Mike Cortese, Fiammetta Cosci, Nelson Cowan, Jarret Crawford, Eveline A. Crone, John Curtin, Randall Engle, Simon Farrell, Pasco Fearon, Mark Fichman, Willem Frankenhuis, Alexandra M. Freund, M. Gareth Gaskell, Roger Giner-Sorolla, Don P. Green, Robert L. Greene, Lisa L. Harlow, Fernando Hoces de la Guardia, Derek Isaacowitz, Janet Kolodner, Debra Lieberman, Gordon D. Logan, Wendy B. Mendes, Lea Moersdorf, Brendan Nyhan, Jeffrey Pollack, Christopher Sullivan, Simine Vazire, Eric-Jan Wagenmakers

**Affiliations:** 1 ELTE, Eotvos Lorand University, Budapest, Hungary; 2 University of Amsterdam, Amsterdam, Netherlands; 3 University of Southern California, Los Angeles, CA USA; 4 Cardiff University, Cardiff, UK; 5 Columbia University, New York, NY USA; 6 University of Wisconsin-Madison, Madison, WI USA; 7 Stanford University, Stanford, CA USA; 8 Maastricht University, Maastricht, Netherlands; 9 Nature Human Behaviour, Springer Nature, London, UK; 10 Emory University, Atlanta, GA USA; 11 University of Melbourne, Melbourne, Victoria Australia; 12 University of Victoria, Saanich, British Columbia Canada; 13 University of Bristol, Bristol, UK; 14 University of New South Wales, Sydney, New South Wales Australia; 15 University of California San Diego, San Diego, CA USA; 16 University College London, London, UK; 17 University of Illinois, Chicago, IL USA; 18 Tilburg University, Tilburg, Netherlands; 19 Rotman Research Institute, Baycrest, Toronto, Ontario Canada; 20 University of Lausanne, Lausanne, Switzerland; 21 Ohio State University, Columbus, OH USA; 22 University of Münster, Münster, Germany; 23 University of North Carolina at Charlotte, Charlotte, NC USA; 24 University of Texas at Austin, Austin, TX USA; 25 Old Dominion University, Norfolk, VA USA; 26 University of California Davis, Davis, CA USA; 27 Georgia Southern University, Statesboro, GA USA; 28 University of Modena-Reggio Emilia, Modena, Italy; 29 University of Massachusetts, Boston, Boston, MA USA; 30 Michigan State University, East Lansing, MI USA; 31 University of Massachusetts, Amherst, Amherst, MA USA; 32 Royal College of Surgeons in Ireland, Dublin, Ireland; 33 University of Nebraska Omaha, Omaha, NE USA; 34 University of Florence, Florence, Italy; 35 University of Missouri, Columbia, MO USA; 36 The College of New Jersey, Ewing Township, NJ USA; 37 Leiden University, Leiden, Netherlands; 38 Georgia Institute of Technology, Atlanta, GA USA; 39 University of Western Australia, Perth, Western Australia Australia; 40 Carnegie Mellon University, New York, NY USA; 41 Radboud University, Nijmegen, Netherlands; 42 University of Zurich, Zurich, Switzerland; 43 University of York, York, UK; 44 University of Kent, Kent, UK; 45 Case Western Reserve University, Cleveland, OH USA; 46 University of Rhode Island, Providence, RI USA; 47 University of California, Berkeley, Berkeley, CA USA; 48 Northeastern University, Boston, MA USA; 49 Boston College, Boston, MA USA; 50 University of Miami, Coral Gables, FL USA; 51 Vanderbilt University, Nashville, TN USA; 52 University of California, San Francisco, San Francisco, CA USA; 53 University of Michigan, Ann Arbor, MI USA; 54 North Carolina State University, Raleigh, NC USA; 55 University of Cincinnati, Cincinnati, OH USA

**Keywords:** Scientific community, Social sciences

Correction to: *Nature Human Behaviour* 10.1038/s41562-019-0772-6, published online 2 December 2019.

In the version of this article initially published, the surname of author Marcus Munafò was misspelled. The error has been corrected in the HTML and PDF versions of the article.

