# Supplementary material for: A consensus-based transparency checklist
Source: Nat Hum Behav. 2019 Dec 2;4(1):4–6. doi: 10.1038/s41562-019-0772-6 (PMC8324470; doi:10.1038/s41562-019-0772-6)
Supplement: Supplementary file 1 — Supplementary Methods, Supplementary Figure 1, Supplementary Table 1. [file 41562_2019_772_MOESM1_ESM.pdf]

nature human behaviour

SUPPLEMENTARY INFORMATION
https://doi.org/10.1038/s41562-019-0772-6

In the format provided by the authors and unedited.

A consensus-based transparency checklist

Balazs Aczel[1*], Barnabas Szaszi[1], Alexandra Sarafoglou[2], Zoltan Kekecs[1], Šimon Kucharský[2], Daniel Benjamin[3], Christopher D. Chambers[4], Agneta Fisher[2], Andrew Gelman[5], Morton A. Gernsbacher[6], John P. Ioannidis[7], Eric Johnson[5], Kai Jonas[8], Stavroula Kousta[9], Scott O. Lilienfeld[10,11], D. Stephen Lindsay[12], Candice C. Morey[4], Marcus Munafò[13], Benjamin R. Newell[14], Harold Pashler[15], David R. Shanks[16], Daniel J. Simons[17], Jelte M. Wicherts[18], Dolores Albarracin[17], Nicole D. Anderson[19], John Antonakis[20], Hal R. Arkes[21], Mitja D. Back[22], George C. Banks[23], Christopher Beevers[24], Andrew A. Bennett[25], Wiebke Bleidorn[26], Ty W. Boyer[27], Cristina Cacciari[28], Alice S. Carter[29], Joseph Cesario[30], Charles Clifton[31], Ronán M. Conroy[32], Mike Cortese[33], Fiammetta Cosci[34], Nelson Cowan[35], Jarret Crawford[36], Eveline A. Crone[37], John Curtin[6], Randall Engle[38], Simon Farrell[39], Pasco Fearon[16], Mark Fichman[40], Willem Frankenhuis[41], Alexandra M. Freund[42], M. Gareth Gaskell[43], Roger Giner-Sorolla[44], Don P. Green[5], Robert L. Greene[45], Lisa L. Harlow[46], Fernando Hoces de la Guardia[47], Derek Isaacowitz[48], Janet Kolodner[49], Debra Lieberman[50], Gordon D. Logan[51], Wendy B. Mendes[52], Lea Moersdorf[42], Brendan Nyhan[53], Jeffrey Pollack[54], Christopher Sullivan[55], Simine Vazire[26] and Eric-Jan Wagenmakers[2]

[1]ELTE, Eotvos Lorand University, Budapest, Hungary. [2]University of Amsterdam, Amsterdam, Netherlands. [3]University of Southern California, Los Angeles, CA, USA. [4]Cardiff University, Cardiff, UK. [5]Columbia University, New York, NY, USA. [6]University of Wisconsin-Madison, Madison, WI, USA. [7]Stanford University, Stanford, CA, USA. [8]Maastricht University, Maastricht, Netherlands. [9]Nature Human Behaviour, Springer Nature, London, UK. [10]Emory University, Atlanta, GA, USA. [11]University of Melbourne, Melbourne, Victoria, Australia. [12]University of Victoria, Saanich, British Columbia, Canada. [13]University of Bristol, Bristol, UK. [14]University of New South Wales, Sydney, New South Wales, Australia. [15]University of California San Diego, San Diego, CA, USA. [16]University College London, London, UK. [17]University of Illinois, Chicago, IL, USA. [18]Tilburg University, Tilburg, Netherlands. [19]Rotman Research Institute, Baycrest, Toronto, Ontario, Canada. [20]University of Lausanne, Lausanne, Switzerland. [21]Ohio State University, Columbus, OH, USA. [22]University of Münster, Münster, Germany. [23]University of North Carolina at Charlotte, Charlotte, NC, USA. [24]University of Texas at Austin, Austin, TX, USA. [25]Old Dominion University, Norfolk, VA, USA. [26]University of California Davis, Davis, CA, USA. [27]Georgia Southern University, Statesboro, GA, USA. [28]University of Modena-Reggio Emilia, Modena, Italy. [29]University of Massachusetts, Boston, Boston, MA, USA. [30]Michigan State University, East Lansing, MI, USA. [31]University of Massachusetts, Amherst, Amherst, MA, USA. [32]Royal College of Surgeons in Ireland, Dublin, Ireland. [33]University of Nebraska Omaha, Omaha, NE, USA. [34]University of Florence, Florence, Italy. [35]University of Missouri, Columbia, MO, USA. [36]The College of New Jersey, Ewing Township, NJ, USA. [37]Leiden University, Leiden, Netherlands. [38]Georgia Institute of Technology, Atlanta, GA, USA. [39]University of Western Australia, Perth, Western Australia, Australia. [40]Carnegie Mellon University, New York, NY, USA. [41]Radboud University, Nijmegen, Netherlands. [42]University of Zurich, Zurich, Switzerland. [43]University of York, York, UK. [44]University of Kent, Kent, UK. [45]Case Western Reserve University, Cleveland, OH, USA. [46]University of Rhode Island, Providence, RI, USA. [47]University of California, Berkeley, Berkeley, CA, USA. [48]Northeastern University, Boston, MA, USA. [49]Boston College, Boston, MA, USA. [50]University of Miami, Coral Gables, FL, USA. [51]Vanderbilt University, Nashville, TN, USA. [52]University of California, San Francisco, San Francisco, CA, USA. [53]University of Michigan, Ann Arbor, MI, USA. [54]North Carolina State University, Raleigh, NC, USA. [55]University of Cincinnati, Cincinnati, OH, USA. *e-mail: aczel.balazs@ppk.elte.hu

Supplementary Materials for

A Consensus-Based Transparency Checklist

This PDF file includes:

Supplementary Methods

Supplementary Methods

Overview

To ensure that the transparency checklist contains most elements that the scientific community considers relevant to the transparency and accountability of behavioural research, we adopted a 'reactive-Delphi' expert consensus process[10; see Fig. S1 for a schematic representation]. The items were evaluated and rated by social and behavioural science journal editors and open science advocates. The Transparency Checklist was modified (items were added, abandoned or reworded, answer options were restructured) until a sufficiently high level of convergence was reached. The expert consensus procedure consisted of two stages. In the first stage, an expert panel was recruited and consulted about the checklist items. Then, journal editors were contacted and asked to rate and comment on the items in two rounds. In stage two, the checklist was finalized using those items that reached consensual support (Supplementary Figure 1). The procedure is described in detail below.

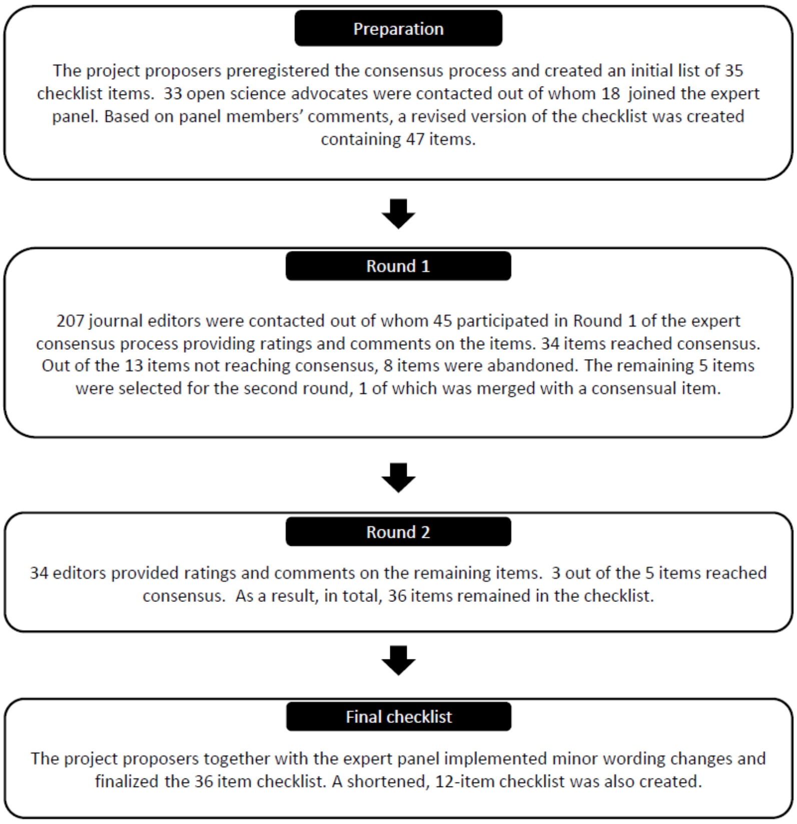

Supplementary Figure 1: Overview of the expert consensus process.

Procedure

Preparation

Preregistering the project

Before the start of the project, on 2018-04-18, a research plan was compiled and uploaded to a time-stamped repository at https://osf.io/v5p2r/registrations/.

Creating the initial checklist items

Before the expert consensus process, the project proposers (the first four authors and the last author; henceforth: proposers) created an initial list of items for the Transparency Checklist after brainstorming and reviewing existing checklists and guidelines with a similar purpose. The initial 35 checklist items are available at https://osf.io/x3g2t/.

Expert consensus procedure

Stage 1

Recruiting panel members

Following this, the proposers contacted 33 open-science advocates from the field of behavioural and social sciences. The selection of these experts was based on our knowledge of the publications in which they propagated openness and transparency of science. These researchers were invited to participate in the further development of the initial list of items and, at the end of the project, the final revision of the checklist. Upon joining this panel, the experts received an online survey (available at https://osf.io/y29a5/).

Consulting the panel members

Following our inquiry, 18 experts joined our panel (henceforth panel members) and provided comments to the initial checklist items (their names are available at https://osf.io/zj6dr/). The proposers discussed each comment and compiled a new version of the checklist on a rolling basis, that is, the panel members who joined later in time reviewed an improved version of the checklist. Based on the panel comments, a revised version of the checklist was created and its 47 items were assessed in the Consensus Design survey, Round 1 (see below).

Recruiting editors for the expert consensus procedure

In order to recruit members for the expert consensus process, we collected email addresses from journal editors using the following procedure. First, we selected the journals with ‘Psychology’ subject in the Scimago (scimagoojr.com) portal (1,075 journals). Psychology label in Scimago seemed to be a convenient searchword for finding journals publishing human behavioural and social empirical studies. We aimed to work with the editors of the high ranked journals, assuming that these journals hold high methodological standards, which makes their editors competent to address questions related to transparency. To this aim, we selected the top 10% of these journals,

using the Scimago Journal Rank (SJR) indicator of 2018 (107 journals). As the checklist was aimed to assist studies reporting primary empirical data, we excluded all journals with 'Review', 'Trends' and 'Perspectives' in their title. Then, we read the policy of the remaining journals and omitted those that do not publish empirical research, leaving a total of 63 journals. The list of the remaining journals is available at https://osf.io/54pzb/. Beside 'Psychology', these journals were associated with the following 79 labels: Accounting, Aging, Anthropology, Applied Mathematics, Arts and Humanities (miscellaneous), Artificial Intelligence, Automotive Engineering, Behavioral Neuroscience, Business and International Management, Business, Management and Accounting (miscellaneous), Cellular and Molecular Neuroscience, Demography, Cognitive Neuroscience, Cognitive Neuroscience, Communication, Computer Science Applications, Cultural Studies, Decision Sciences (miscellaneous), Development, Developmental Biology, Developmental Neuroscience, Developmental Neuroscience, Ecology, Evolution, Behavior and Systematics, Economics and Econometrics, Education, Environmental Science (miscellaneous), Epidemiology, Gender Studies, Genetics, Geriatrics and Gerontology, Gerontology, Health (social science), History and Philosophy of Science, Human-Computer Interaction, Infectious Diseases, Information Systems, Information Systems and Management, Language and Linguistics, Law, Life-span and Life-course Studies, Management of Technology and Innovation, Marketing, Marketing, Medicine (miscellaneous), Neurology, Neurology (clinical), Neuroscience (miscellaneous), Neuroscience (miscellaneous), Nutrition and Dietetics, Oncology, Organizational Behavior and Human Resource Management, Pathology and Forensic Medicine, Pediatrics, Perinatology and Child Health, Philosophy, Physical Therapy, Sports Therapy and Rehabilitation, Physiology, Physiology (medical), Political Science and International Relations, Psychiatric Mental Health, Psychiatry and Mental Health, Psychiatry and Mental Health, Public Health, Environmental and Occupational Health, Religious Studies, Social Sciences (miscellaneous), Social Work, Sociology and Political Science, Speech and Hearing, Sports Science, Statistics and Probability, Strategy and Management, Toxicology, Transportation, Visual Arts and Performing Arts.

We retrieved the contact information of the chief editors and two associate editors from each journal (189 contacts). When there were more than two associate editors in an editorial board, we picked two names without any systematic method. Finally, we extended the contact list by adding 18 experts from other behavioral sciences (e.g., management science, political science). We selected these experts after a non-systematic literature review suggesting these individuals to be particularly engaged in transparency and open science issues. The resulting list contained the contact details of 207 journal editors and experts. Although the resulting list contained 6 non-editor experts, for the sake of simplicity, we call this group 'editors'.

Round 1

Consulting the editors

We emailed 207 editors and invited them to participate in the consensus process. Although we had questions in the survey referring to their role at their journal, we did not ask them explicitly to answer the survey as a journal editor. (The text of our invitation is available at

https://osf.io/8b52k/). The consensus design survey was sent out individually to everyone on our contact list between 2018-12-14 and 2018-12-18. The participants received the question in a Google Forms Survey.

The survey contained the proposed checklist items along with their answer options. The items covered four components of a study: *Preregistration; Methods; Results and Discussion; Data, Code, and Materials Availability.* Noted that the boundaries of these labels can vary among research fields and for some researchers certain items might better fit under another label.

The respondents could rate each item on a 9-point Likert-type ‘recommendation scale’ ranging “Definitely do not include this item” to “Definitely include this item”. Following each section, the respondents could leave comments regarding the items. The survey is available at https://osf.io/ptjcz/.

Evaluating the survey results

We elicited responses from 45 editors (one of them was a member of the panel as well) between 2018-12-14 and 2019-02-02 (their list is available at https://osf.io/zj6dr/). Next, the proposers of the project amended and modified the checklist using the collected comments and recommendations. The survey-answers are available at https://osf.io/43hxk/. As described in the preregistration plan, if the interquartile range of the ratings of an item was 2 or smaller on the recommendation scale, we regarded that particular item as having reached consensus. We concluded that support was obtained on a consensus item if the median of its rating was 6 or higher. In this round, 34 out of the 47 items reached consensus. Following our preregistered plan, the proposers of the project discussed the comments and recommendations collected in this round and decided which items to select for the next survey. As a result, out of the 13 items not reaching consensus, 8 items were abandoned. For an in-depth description of all exclusion criteria, we refer the interested reader to our online summary of results. The remaining 5 items were selected for the second round, 1 of which was merged with a consensual item. No other alteration or rephrasing took place in the content of the checklist. The explanations for each excluded item are provided at https://osf.io/zyed8/ and the summary table of the remaining items are available in Supplementary Table 1.

Supplementary Table 1

Summary Table of the Statistics and the Texts of the Final Checklist Items

Stage 1 Round 1						Stage 1 Round 2						Stage 2
**Section**	**Item**	**rating mean**	**rating median**	**rating IQR**	**Consensus**	**Item**	**rating mean**	**rating median**	**rating IQR**	**Consensus**	**Exclusion**	**wording adjustments**
**Preregistration**	(1) data analysis plan (Yes | No | N/A)	7.84	9	2	Yes							Prior to analyzing the complete data set, a time-stamped preregistration was posted in an independent, third-party registry for the data analysis plan.
	(2) The manuscript includes a URL to all preregistrations that concern the present study. (conditional on anything preregistered) (Yes | No | N/A)	7.89	9	2	Yes							
	(3) The study was preregistered ...before any data were collected (etc Dropdown)	8.36	9	1	Yes							
	The preregistration fully describes …<br><br>(4a) all participant eligibility criteria (e.g., fluent English speakers) (Yes | No | N/A)	8.07	9	2	Yes (merged with next item)							
	(4b) [appears if the previous item is confirmed] all operationalizations of the participant eligibility criteria (e.g., cutoff scores in a language test) (Yes | No | N/A)	7.09	8	3	No	all inclusion and exclusion criteria for participation (e.g., English speakers who achieved a certain cutoff score in a language test) (Yes | No | N/A)	8.50	9.00	1.00	Yes	No	
	(5) all procedures for assigning participants to conditions (Yes | No | N/A)	8.13	9	1	Yes							
	(6) all procedures for randomizing stimulus materials (Yes | No | N/A)	7.69	9	2	Yes							
	(7) all procedures for ensuring that participants, experimenters, and data-analysts were kept naive to potentially biasing information (Yes | No | N/A)	7.11	9	3	No	all procedures for ensuring that participants and experimenters were kept naive (blinded) to potentially biasing information (Yes | No | N/A)	8.06	9.00	1.00	Yes	No	any procedures for ensuring that participants, experimenters, and data-analysts were kept naive to potentially biasing information
	(8) a rationale for the sample size used (e.g., an a priori power analysis) (Yes | No | N/A)	7.78	9	2	Yes							
	(9) your measures of interest (e.g., friendliness) (Yes | No | N/A)	8.18	9	1	Yes							the measures of interest (e.g., friendliness).
	(10) [appears if the previous item is confirmed] all operationalizations	7.82	9	1	Yes							

	for your measures of interest (e.g., a questionnaire measuring friendliness) (Yes | No | N/A)											
	(11) how the data were planned to be preprocessed (e.g., transformed, cleaned, normalized, smoothed) (Yes | No | N/A)	8.11	9	1	Yes							the data preprocessing plans (e.g., transformed, cleaned, normalized, smoothed).
	(12) how missing data (e.g., dropouts) were planned to be handled(Yes | No | N/A)	8.27	9	1	Yes							
	(13) the intended statistical analysis for each research question (This may require, for example, information about the sidedness of the tests, inference criteria, corrections for multiple testing, model selection criteria, prior distributions etc.) (Yes | No | N/A)	8.13	9	1	Yes							the intended statistical analysis for each research question (this may require, for example, information about the sidedness of the tests, inference criteria, corrections for multiple testing, model selection criteria, prior distributions etc.).
**Method**	(14) the rationale for the sample size used (e.g., an a priori power analysis) (Yes | No | N/A)	8.02	9	1	Yes							
	(15) how participants were recruited (Yes | No | N/A)	8.40	9	0	Yes							
	(16) how participants were selected (e.g., eligibility criteria) (Yes | No | N/A)	8.53	9	0	Yes							
	(17) what compensation was offered for participation (Yes | No | N/A)	8.11	9	1	Yes							
	(18) how participant dropout was handled (e.g., replaced, omitted, etc) (Yes | No | N/A)	8.58	9	0	Yes							
	(19) how participants were assigned to conditions (Yes | No | N/A)	8.67	9	0	Yes							
	(20) how stimulus materials were randomized (Yes | No | N/A)	8.36	9	1	Yes							
	(21) how participants, experimenters, and data-analysts were kept naive to potentially biasing information (Yes | No | N/A)	8.09	9	1	Yes							whether (and, if so, how) participants, experimenters, and data-analysts were kept naive to potentially biasing information
	(22) the study design, procedures, and materials to allow independent replication (Yes | No | N/A)	8.62	9	0	Yes							
	(23) your measures of interest (e.g., friendliness) (Yes | No | N/A)	8.69	9	0	Yes							the measures of interest (e.g., friendliness).
	(24) [appears if the previous item is confirmed] all operationalizations for your measures of interest (e.g., a questionnaire measuring friendliness) (Yes | No | N/A)	8.49	9	0	Yes							
	(25) any changes to the preregistration (such as changes in eligibility criteria, group membership cutoffs, or experimental procedures)? (conditional on preregistration) (Yes | No | N/A)	8.73	9	0	Yes							

	(26) distinguishes sharply between "confirmatory" (i.e., prespecified) and "exploratory" (i.e., not prespecified) analyses (Yes | No | N/A)	8.33	9	0	Yes							distinguishes explicitly between "confirmatory" (i.e., prespecified) and "exploratory" (i.e., not prespecified) analyses
	(27) describes how violations of statistical assumptions were handled (Yes | No | N/A)	7.56	8	2	Yes							
**Results and Discussion**	(28) justifies all statistical choices (e.g., including or excluding covariates; applying or not applying transformations; use of multi-level models vs. ANOVA) (Yes | No | N/A)	7.89	9	2	Yes							
	(29) reports the sample size for each cell of the design (Yes | No | N/A)	8.62	9	0	Yes							
	(30) reports how incomplete or missing data were handled (Yes | No | N/A)	8.69	9	0	Yes							
	(31) presents protocols for data preprocessing (e.g., cleaning, discarding of cases and items, normalizing, smoothing, artifact correction) (Yes | No | N/A)	8.36	9	1	Yes							
	The following have been made publicly available … (32) The (processed) data, on which the analyses of the manuscript were based (Yes | No | N/A)	7.73	9	3	No	(42) the (processed) data, on which the analyses of the manuscript were based (Yes | No | N/A)	8.00	9.00	1.75	Yes	No	
	(33) all code and software (that is not copyright protected) (Yes | No | N/A)	7.96	9	2	Yes							
	(34) all instructions, stimuli, and test materials (that are not copyright protected) (Yes | No | N/A)	7.93	9	2	Yes							
**Openness**	(35) Are the data properly archived (i.e., would a graduate student with relevant background knowledge be able to identify each variable and reproduce the analysis)? (conditional on anything shared) (Yes | No | N/A)	7.93	9	2	Yes							
	(36) The manuscript includes a statement concerning the availability and location of all research items, including data, materials, and code relevant to your study (such as the TOP statement by Aalbersberg et al., 2018; https://osf.io/sm78t/). (Yes | No | N/A)	8.02	9	1	Yes							The manuscript includes a statement concerning the availability and location of all research items, including data, materials, and code relevant to the study.

Round 2

Consulting the editors

For this round, a new rating survey was created with the 5 remaining items along with reflections to the received comments and the aggregated ratings that each item received in Round 1. Only those 43 editors who responded to the first round and indicated that we can get in contact again received the survey. Two editors did not wish to be contacted with new versions of the checklist. The survey is available at https://osf.io/5xh7d/.
We received 34 completed surveys between 25/02/2018 and 11/03/2019. The survey-answers are available at https://osf.io/y6457/. After analysing the collected data, 2 items were dropped from the final survey as the ratings failed to show consensus as the interquartile range of the recommendation ratings was greater than 2. As a result of Round 2, 36 items remained in the checklist. A summary of the round two is available at https://osf.io/zyed8/.

Furthermore, we also explored the differences in the item ratings between subdisciplines. We used the following categories: Applied Psychology, Clinical and Personality Psychology, Developmental and Educational Psychology, Experimental and Cognitive Psychology, Neuropsychology and Physiological Psychology, Social Psychology, Other Psychology, Other Behavioral Science. To do this, we calculated the median ratings for each subdiscipline and calculated the interquartile range based on these values across subdisciplines. We found that the interquartile range was greater than 2 for only two items (item 17 and 36) (results are available at https://osf.io/gsd3u/). Therefore, we did not create discipline-specific checklists or checklist-sections.

As a final step, we created a user-friendly ShinyApp by which the authors can easily answer the checklist questions, leave comments, and generate a report that contains their checklist answers along with the metadata of their manuscript. In addition, the proposers created a shortened, 12-item long version of the checklist as well, containing the most essential items (http://www.shinyapps.org/apps/TransparencyChecklist/ and http://www.shinyapps.org/apps/ShortTransparencyChecklist/ for the shortened, 12-item version). For this admittedly subjective selection, the proposers tried to choose those items that appeared most important for transparency. Their judgment was based on the item ratings and the comments collected during the consensus procedure. As a pilot test, we asked 6 researchers to fill out the 36-item checklist regarding their most recent empirical study. On average, it took them 6 minutes (range: 3-15) to respond to the items.

Stage 2

In Stage 2, the proposers wrote the manuscript, including the present supplement on the basis of descriptive data and free text responses gathered in Stage 1 along with a suggested final list of checklist items. All texts and materials were sent to the panel members and all contributing editors. Each contributor was encouraged to provide feedback on the manuscript, the report, and the suggested final checklists. After all discussions, minor wording changes were implemented for item 1, 9, 11, 13, 23, and 36. No contributor objected to the content and form of the submitted materials and all approved the final item list.

Notable deviations from the preregistration

The project was preregistered on: https://osf.io/v5p2r/registrations. One deviation from our original plan was that we have involved the panel members in the discussion of the items from the very start of the project and not just in the final stage. We slightly changed the wording of the survey questions to increase comprehension. Also, we excluded some low rated items from the consensus process after Round 1. Justifications for our exclusions are added to the summary table of the results, available at https://osf.io/zyed8/. Furthermore, we did not take panel meeting minutes that we planned to share.
